# Whole-Blood Cellular Responses: A Promising Indicator of SARS-CoV-2 Immunity Compared to Serology

**DOI:** 10.3390/jcm14196889

**Published:** 2025-09-29

**Authors:** Lucas M. Zhou, Elizabeth H. Duncan, Rupsa C. Boelig, Margaret Costanzo, Jeffrey R. Currier, Elke S. Bergmann-Leitner

**Affiliations:** 1 Immunology Core, Biologics Research & Development, Walter Reed Army Institute of Research—WRAIR, Silver Spring, MD 20910, USA; lucaszhou888@gmail.com (L.M.Z.); elizabeth.h.duncangooden.civ@health.mil (E.H.D.); 2 Division of Maternal Fetal Medicine, Department of Obstetrics and Gynecology, Sidney Kimmel Medical College, Thomas Jefferson University Hospital, Philadelphia, PA 19107, USA; rupsa.boelig@jefferson.edu; 3 Emerging Infectious Diseases Branch, Walter Reed Army Institute of Research—WRAIR, Silver Spring, MD 20910, USA; margaret.costanzo@gmail.com; 4 Viral Diseases Branch, Walter Reed Army Institute of Research—WRAIR, Silver Spring, MD 20910, USA; jeffrey.r.currier.civ@health.mil

**Keywords:** immunity, SARS-CoV-2, T cell responses, cytokines, multiplex testing platform, antigens SARS-CoV-2, COVID-19

## Abstract

**Background:** Currently available immunological tests for SARS-CoV-2 assess only antibody responses. Despite the growing evidence that T cells play a crucial role in protection, especially against emerging viral variants, no routine test is available to determine T cell immunity. The prognostic value of SARS-CoV-2-specific antibodies for determining whether individuals are immune and protected against disease remains uncertain. This is in part due to the following: (a) specificity and limitations such as the sensitivity of antibody tests, and (b) the lack of correlation between antibody titers (quantity) and the antiviral function of antibodies (quality). Approximately a quarter of SARS-CoV-2-infected patients with symptoms fail to show seroconversion in serological assays. **Methods**: The current report describes the development and application of a whole-blood-based assay to detect previous exposure to SARS-CoV-2. Whole blood is stimulated with SARS-CoV-2-derived peptides identified during assay development and stimulation-induced cytokines quantified using a multiplex testing platform. The resulting cytokine profiles are generated using computational tools to identify previous exposure to the virus. **Results**: The application of the assay revealed a lack of self-awareness of individuals’ COVID-19 infection history and demonstrated the value of this new assay to assess the prevalence of SARS-CoV-2 exposure history and immunity in populations. **Conclusions**: Positive responses in this assay can facilitate the identification of underlying causes of unexplained symptoms and provide clinically actionable insights for healthcare applications, including in the continued conundrum of post-acute sequela of SARS-CoV-2 infection (PASC or “long COVID”), for which both diagnosis and management remain challenging.

## 1. Introduction

The objective of the present study was to develop a sensitive blood test that assesses cellular immunity and previous exposure to SARS-CoV-2. The need for such an assay has been discussed extensively by the research community as discordance between PCR results (detecting virus particles in mucosal tissues) and serological results (measuring antibodies to SARS-CoV-2 in the blood) has been reported [1]. In contrast, a recent study reported that positive PCR results correlated with 98% of positive SARS-CoV-2-specific T cell responses while only 83% of the individuals had measurable antibody responses in the acute phase of infection [2]. Measuring T cell responses eight weeks after infection to assess exposure and immunity showed 84% positivity while only 52% of the individuals had measurable antibodies. Recent reports confirm the results of an earlier study that T cell responses are more persistent compared to antibody responses [3,4,5]. Having a sensitive and specific diagnostic T cell assay is imperative to (a) establish threshold responses that correlate with protective immunity, (b) determine the immune status of individuals, (c) identify high-risk individuals, especially immunocompromised patients [6], and (d) conduct clinical research on the phenotyping of COVID-19 infections that predispose individuals to developing PASC and to a clinical diagnosis of PASC, a persistent condition that impacts all age groups even in the setting of “milder” variants and broad immunity [3,7,8,9].

Five years into the pandemic, a valid question is whether there is still the need for a new T cell-based blood test to assess immunity. Early on in the pandemic, the Food and Drug Administration (FDA) approved “T-Detect”, an assay that sequences the T cell receptors to determine the presence of SARS-CoV-2-specific T cells [10]. This is an agnostic test that fails to report on cell frequencies and fine specificities and can that only report general exposure. Two additional research-use-only (RUO) blood tests are now available, namely *T.Spot.COVID* (Oxford Immunotec, [2]) and *Covi-Feron* (SD Biosensors, [11]). Both are based on detecting IFNγ responses in blood after stimulation with a mix of three variant SARS-CoV-2 spike proteins and the SARS-CoV-2 nucleocapsid protein. Both rely on the assumption that the presence of IFNγ-producing T cells specific for the S and N protein reliably correlates with SARS-CoV-2 infection. Monitoring changes in the frequencies of SARS-CoV-2-specific leukocytes producing IFNγ or in the total magnitude of this cytokine response may not provide a clear threshold for determining the previous exposure and persisting protective immunity of the respective donor. A previous study applying an IFNγ release assay to determining the COVID-19 history of blood donors attested to the fact that cutoff values for IFNγ release will continue to change as the seroprevalence and presence of long-term adaptive cellular immunity against SARS-CoV-2 increase [12]. Therefore, assessing the broader SARS-CoV-2-specific cytokine profile in whole blood to assess the immunity and durability of immune responses to SARS-CoV-2 variants might increase the accuracy of cell-based assessment of immunity [13].

The goal of the present work was to establish a blood-based assay that (a) establishes the broad antigen-specificity profile of T cell responses [14] and (b) identifies and subsequently utilizes other T cell factors besides IFN-γ to increase sensitivity and specificity. A recent study concluded that a more sophisticated diagnostic assay than those currently on the market is desirable [15]. Our strategy of profiling T cell responses capable of defining SARS-CoV-2 immunity may address this need. These tools can be applied in research, to better characterize COVID-19 infection and understand the phenotypes that may predispose individuals to severe symptoms or PASC, as well as in clinical care, as the diagnosis of PASC remains a challenge when symptoms present months after a mild or even asymptomatic infection that an individual may be unaware of.

## 2. Materials and Methods

### 2.1. Samples

The first set of experiments were matrix experiments to simultaneously evaluate stimulants and readout factors. Cryopreserved peripheral mononuclear cells (PBMCs) from various sources (commercial sources: Ray Biotech (Norcross, GA, USA), Cellero (Charles River, Memphis, TN, USA), exempt human use (NHSR) protocols based on IRB-approved blood collection protocols: RV229, WRAIR#2139, WRAIR#2769, WRAIR#2567) and distinct histories of COVID-19 exposure were included in these experiments (Table 1). The demographics of the study participants reflect a distribution of age and ethnicity reflective of U.S. military service members (18–49 years, no comorbidities). The investigators adhered to the policies for protection of human subjects as prescribed in AR 70-25. The research did not involve human subjects (NHSR protocols) as the samples used were de-identified and no link between samples and subjects exists.

### 2.2. Isolation of Peripheral Blood Mononuclear Cells (PBMC)

Heparinized blood was centrifuged (300× *g*, 10 min) to remove plasma. Blood cells were resuspended in Hank’s balanced salt solution (HBSS, Quality Biological, Gaithersburg, MD, USA) and PBMC isolated using Ficoll–Hypaque (Cytiva, Uppsala, Sweden) per the manufacturer’s instructions.

### 2.3. PBMC Stimulation

Cryopreserved PBMCs were stimulated with the respective antigens (Table 2) in a 37 °C, 5% CO_2_ incubator for 24 h. The cell viability of thawed PBMCs was >92%, as measured by a Luna-FL™ Dual Fluorescence cell counter (fluorescence protocol with AO/PI to determine cell viability). Culture supernatants were tested using a multiplex electro-chemiluminescence platform (MesoScale Diagnostics (MSD), Gaithersburg, MD, USA) as described below.

### 2.4. Whole Blood Stimulation

Heparinized blood was transferred from the blood collection tube in 1 mL aliquots per well of a 24-well plate (Thermo Fisher Scientific, Waltham, MA, USA). Stimulants (i.e., Spike protein CoV-2 (SCOV2), SARS-CoV-2 nucleocapsid (COVN), AP3a, ORF3a, *CoV-2 membrane protein*, and a megapool of non-structural proteins (NSPX) for the testing stimulation, HA as the reference stimulation, serum-free culture media as a negative control, and anti-CD3 as a positive control) were added to the respective cultures in volumes less than 50 µL (final concentrations of stimulants Table 2). Cultures were incubated in a 37 °C, 5% CO_2_ incubator for 24 h. Cultures were then collected and centrifuged for 10 min at 300× *g*, and the culture supernatants were collected for cytokine analysis using the MSD platform.

### 2.5. Cytokine Measurement

Profiling of stimulation-induced chemokines/cytokines was conducted using an electro-chemiluminescence based multiplex testing platform (Mesoscale Discovery (MSD), Gaithersburg, MD, USA). The complete test panel consisted of the following: IFN-γ, bFGF, C-reactive protein, Eotaxin, Eotaxin-3, Flt-1, GM-CSF, ICAM-1, IL-10, IL-12/IL-23, IL-12p70, IL-13, IL-15, IL-16, IL-17A, IL-17AGenB, IL-17A/F, IL-17B, IL-17C, IL-17D, IL-1RA, IL-1 α, IL-1β, IL-2, IL-21, IL-22, IL-23, IL-27, IL-3, IL-31, IL-4, IL-5, IL-6, IL-7, IL-8, IL-8(HA), IL-9, IP-10, MCP-1, MCP-4, MDC, MIP-1α, MIP-1β, MIP-3α, PIGF, SAA, TARC, Tie-2, TNF-α, TNF-β, TSLP, VCAM-1, VEGF, VEGF-C, and VEGF-D. The quantification of factors was performed according to the manufacturer’s protocol. Briefly, sera were diluted in MSD diluent in a ratio of 1:2 and then added to the multiplex plates. After 2 hrs of incubation at RT, the plates were washed with MSD wash buffer, and Sulfotag-conjugated detection antibodies were added. Finally, the plates were washed with MSD wash buffer, MSD substrate buffer was added and the plates were read using the MSD reader QuickPlex SQ120 (Rockville, MD, USA). Data were expressed as pg/mL based on recombinant cytokine standard curves [16,17]. Initial data analysis including the determination of factor concentrations was performed with the MSD WorkbenchTM Version 4.0 software (MesoScale Discovery, Rockville, MD, USA).

### 2.6. Serological Testing

The MSD V-PLEX platform was used, in the form of 10-plex assays, utilizing pre-printed antigens (HA-trimer Influenza A (Hong Kong H3), spike trimers for SARS-CoV-2, SARS-CoV-1, MERS-CoV, and beta-coronaviruses HKU-1 and OC43, spike N-terminal domain (NTD), receptor binding domain (RBD), and nucleocapsid protein (COVN) for SARS-CoV-2, and bovine serum albumin (BSA) as a control) as previously reported [18]. Seroconversion was assumed if the mean luminescence signal to COVN exceeded the cutoff, defined as the mean + 2 standard deviations of the pre-pandemic samples. All serological data were expressed as log10-transformed mean luminescence signals.

### 2.7. Statistical Analysis

Univariate analysis was conducted using stimulation indices, i.e., dividing the mean cytokine response after stimulation by the negative control (response to media alone). Two-sample tests were used to determine differences between groups, employing the Bonferroni correction to adjust for multiple comparisons.

To demonstrate changes in the fine specificity of the CoV-2-specific response, we chose to visualize the responses as pie charts (raw data are shown in Supplementary Figure S4). For the comparison of antigen-specific responses between groups, we treated each pair of pie charts as a set of 2 × 2 contingency tables. For a given cytokine (row), we selected the same antigen (slice) in both charts and compared their proportions. Differences in proportions were tested using a two-proportion Z-test with a significance level of α = 0.05. To correct for multiple testing, we applied a Bonferroni adjustment (α = 0.05/18), accounting for 9 antigen comparisons per cytokine across 2 cytokines (9 × 2 = 18 total comparisons).

The agreement in CoV-2-specific cytokine responses between PBMC and whole-blood cultures was determined by regressing log10-transformed data. Random Forest models (based on the R *caret* package) were used to create a model that could predict COVID infection status. We established a set of criteria to guide predictor selection, choosing the combination that (1) achieved the highest accuracy across the largest number of stimulus combinations and trials, and (2) included the minimal number of predictors necessary. This approach ensured the robust, reproducible selection of predictive features while avoiding arbitrary cutoffs. An initial model was trained on a limited dataset using the ranger method, with *mtry* set to the rounded down square root of the number variables, split rule set to the *Gini Impurity*, and *min.node.size* set to 1. To test the predictive accuracy of this approach, leave-one-out-cross-validation (LOOCV) was performed, where one subject was removed from the dataset and the remaining data was used to train the model and then predict whether the excluded subject had COVID-19. The *varImp* function was used to determine the variable importance, and the average variable importance was reported to assess the contribution of each cytokine toward the model’s ability to detect COVID-19 infection. The full Random Forest model was trained using the ranger method with the same parameters as above. The predictive accuracy of the model was evaluated using the *repeated cv* method, sub-sampling the data 5-fold and resampling ten times. All statistical analysis was performed in the R programming language utilizing the *corr*, *caret*, *rstatix* packages. Visualization was performed using the *fmsb*, *ggpubr*, *pheatmap*, *ggrepel,* and *corrplot* packages.

## 3. Results

### 3.1. Cytokine Profiles Induced by SARS-CoV-2 Antigens

**Figure 1:**
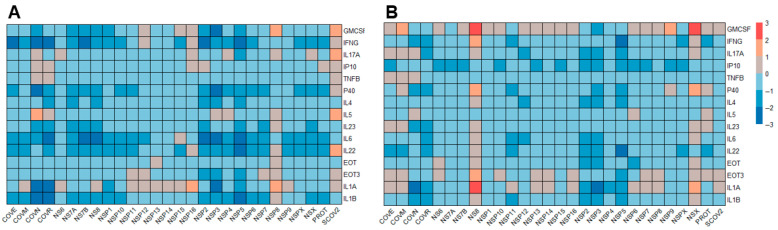
Identification of net cytokine responses significantly increased after stimulation of PBMC with different SARS-CoV-2 antigens (X axis). Heatmaps are visualizing the log-transformed mean net cytokine responses (normalized based on pre-pandemic responses). Rows indicate cytokine/chemokine, columns correspond to stimulating antigen) in PBMC collected from immune donors (sample size n = 34) collected in 2021 (Panel (**A**)) or 2024 (Panel (**B**)). Each cell shows the net change in a given cytokine response to the indicated antigen. The color scale represents net changes compared to pre-pandemic responses, with blue indicating values below the mean and red indicating values above the mean (range: −3 to +3). Each cell shows the relative magnitude of a given cytokine response to the indicated antigen.

Initial testing conducted using pandemic samples at different time points during the pandemic to profile the cytokine landscape by measuring 54 factors produced in response to in vitro stimulation identified 15 factors that showed notable cytokine production when stimulated with SARS-CoV-2 antigens in samples obtained from individuals with previous COVID-19 infection (Figure 1).

### 3.2. Comparative SARS-CoV-2-Specific Cytokine Profiling of PBMC Vs. Whole Blood

**Figure 2:**
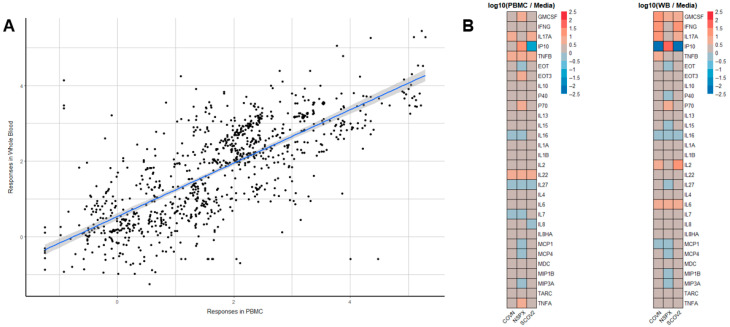
Comparison of SARS-CoV-2-specific cytokine/chemokine responses in whole blood vs. PBMCs from donors with COVID-19 history. (**A**) Agreement in CoV-2-specific cytokine responses between PBMCs and whole-blood cultures. PBMCs vs. whole blood (n = 4 donors) were stimulated with eight antigens (SCOV2, RBD, COVN, AP3a, ORF3a, CoV-2 membrane protein, non-structural proteins peptide pools (NSPX)). Dot represents an individual measurement and the line is a linear regression line with 95% confidence interval (shaded area) (**B**) Heatmaps visualizing the cytokine profile of antigen-specific cytokine responses induced after in vitro stimulation with either COVN, NSPX, or SCOV2. Rows indicate cytokines; columns indicate stimulation. The color scale represents relative expression, with blue indicating values below the mean and red indicating values above the mean (range: −2.5 to +2.5). Each cell shows the relative magnitude of a given cytokine response to the indicated antigen. Data expressed as the stimulation index, i.e., log10 (cytokine response to antigen stimulation divided by the cytokine response in negative control).

The objective of this study was to develop a whole-blood-based assay that can inform on previous exposure to SARS-CoV-2 to determine the level of immunity or persistence of viral reservoirs of SARS-CoV-2. Blood from healthy donors with known vaccination and COVID-19 history was obtained and tested either by stimulating whole blood directly or first isolating PBMCs and then stimulating PBMCs with either a negative control (culture media only), AP3a, ORF3a, SARS-CoV-2 nucleocapsid (COVN), pool of nonstructural proteins (NSPX), or anti-CD3 (positive assay control). Cytokine/chemokine content in culture supernatants (Figure 2) was quantified using an electro-chemiluminescence based multiplex platform (MesoScale Diagnostics) that has an exceptionally wide linear range [19].

Comparing the SARS-CoV-2-specific cytokine landscape stimulated in whole blood vs. PBMCs showed an overall acceptable agreement despite some quantitative differences in these factors (r^2^ = 0.554, Figure 2A). There were, however, some quantitative differences in the responses to either COVN, SCOV, or NSPX depending on whether whole blood or PBMC was tested (Figure 2B). The whole-blood assay demonstrated greater sensitivity and resolution, likely due to its broader dynamic range, which allowed for the detection of both low- and high-level responses without signal saturation.

### 3.3. Identification of Stimulants and Cytokine Readout Method for Highest Accuracy in Determining Previous Exposure

**Figure 3:**
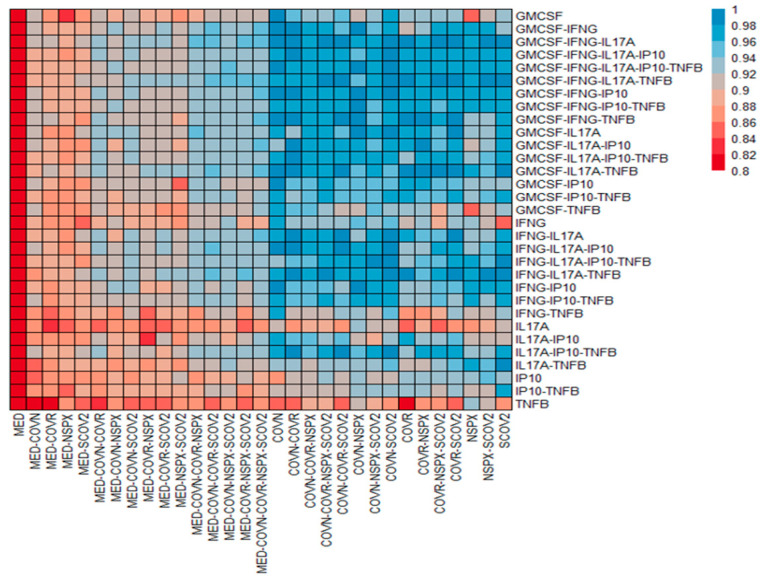
Accuracy of readout combinations in predicting COVID-19 status using Random Forest (RF) modeling. The heatmap visualizes the accuracy of the model created by using data on every combination of stimuli (columns) and analytes (rows). The color scale denotes the RF model’s accuracy, ranging from low accuracy (red, 0.8) to higher accuracy (blue, 1). Data were generated by whole-blood testing of n = 30 healthy donors via a blood collection protocol.

Next, we tested whole-blood samples from healthy donors (n = 30) with known COVID-19 disease and vaccination history to down-select stimulant–readout combinations with the highest signal-to-noise ratio. Random forest modeling was performed to (1) confirm the importance of the selected five factors in predicting the COVID-19 status of donors (Supplementary Figure S1) and (2) determine which of the stimulating antigens and which of the readout methods resulted in the highest accuracy of classifying donors accurately regarding previous exposure/infection with SARS-CoV-2 (Figure 3).

Based on these results, stimulating whole blood with only the nucleocapsid and performing multiplex detection of GM-CSF/IFNγ/IL17A had the highest accuracy (Supplementary Table S1). We did not consider including IL-6 as test cytokine as this cytokine has been used as general inflammation biomarker and may, therefore, have impacted the specificity and sensitivity of the assay.

Going forward, a cellular test to assess SARS-CoV-2 immunity should include nucleocapsids (best predictor for previous infection), spike protein/peptides derived from spike protein (which informs on previous exposure to SARS-CoV-2 spikes in the form of vaccine or infection), a nonstructural protein megapool (NSPX), a negative control (tested here media control), and a positive assay control (tested here anti-CD3).

### 3.4. Vaccination and Disease Status Influence Cytokine Profile

**Figure 4:**
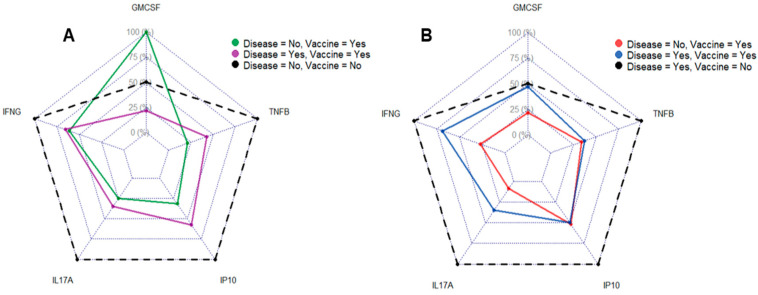
Distinct SARS-CoV-2-specific cytokine profiles reflecting distinct COVID-19 history. Radar plots compare the magnitude of COVN-specific cytokine responses (GM-CSF, TNFβ, IP-10, IL-17A, and IFNγ) based on vaccine and disease history. Cytokine profiles are stratified based on either on self-reported COVID-19 history (Panel (**A**); sample size n = 30)) or COVN seroconversion (Panel (**B**), n = 20). For each analyte, the mean value was calculated within each of the six groups. The highest mean across groups was set to 100%, and all other group means were scaled relative to this maximum. For a given analyte, the 100% reference is the same in both plots, allowing direct comparison. Scale on radar plot indicates the magnitude of the response (e.g., 100% corresponds to highest cytokine concentration measured).

The newly developed multiplex assay was applied to whole-blood samples from 30 healthy donors to establish a cytokine profile associated with vaccination vs. previous infection. The results were then either stratified based on self-reported COVID-19 history (i.e., whether vaccinated and/or previously infected, Figure 4A) or based on seroconversion to COVN (Figure 4B). The profile based on self-reporting showed that the highest IL17A, IFNγ, TNFβ, and IP10 levels were measured in the group that reported no known COVID-19 history. Donors reporting only having received vaccination had the highest GM-CSF response and otherwise similar magnitudes of IFNγ and IL17A responses to the group that reported both, disease and vaccine. Since the comparison of the magnitude of cytokine responses revealed unexpected findings, we sought to confirm the COVID-19 status of the donors by serology against COVN (Supplementary Figure S2).

Replotting the data based on seroconversion showed that individuals that previously had COVID-19 (but thought that they never had an infection) had the highest IL17A, IFNγ, TNFβ, and IP10 response (Figure 4B). The magnitude of GM-CSF responses to COVN was comparable in donors that either had disease or both the disease and a vaccine. In contrast, individuals that only had the vaccine had the lowest GM-CSF, IL17A, and IFNγ response and had comparable amounts of TNFβ and IP10 as donors that had both the disease and a vaccine. Interestingly, having been vaccinated and not having had the disease led to TNFβ and IP10 responses equivalent to those measured in vaccinated donors that previously had the disease. The current data suggest an advantage of establishing cytokine profiles rather than measuring a single cytokine to determine SARS-CoV-2 immunity.

### 3.5. Distinct Serological Profiles of Donors Based on Self-Reported COVID-19 History

From the fact that individuals were unaware of their prior COVID-19 disease, we deducted that those donors likely had an unrecognized prior infection. We established their SARS-CoV-2-specific serological profile stratified based on the assumption that seroconversion to nucleocapsid without reported disease awareness represented a previously unrecognized infection (Supplementary Figure S3). The state of seropositivity for the SARS-CoV-2 nucleocapsid can be explained either as a prior asymptomatic infection (the lack of a clinically confirmed absence of symptoms makes this hard to ascertain) or a recall bias. While no statistical significance was noted between the two groups, there were trends showing that individuals with previous symptomatic disease had lower antibody titers against CoV-2 antigens. In general, donors with previous symptomatic infection had higher variability in antibody titers against SARS-CoV-2 antigens, but also more variability in the cross-reactivity against the spike proteins of SARS-CoV1 and MERS-CoV (Supplementary Table S2). Interestingly, this trend was not observed for responses to spike proteins of the seasonal CoVs HKU-1 and OC43 or the reference analyte (Influenza hemagglutinin). In conclusion, serological profiles appear distinct when comparing individuals with confirmed previous infections to individuals that self-reportedly did not have COVID-19 before.

### 3.6. Cellular and Serological Anti-SARS-CoV-2 Responses Show Only Weak Correlation

**Figure 5:**
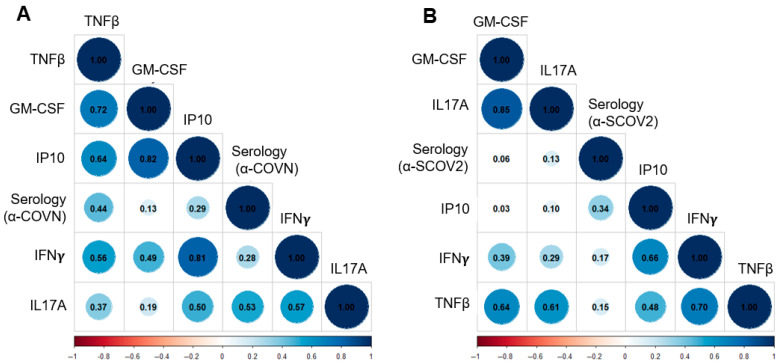
Correlation between serological and cellular responses to SARS-CoV-2. Correlation matrices visualize the functional relationship between SARS-CoV-2-specific serological and cellular responses to SARS-CoV-2 nucleocapsid (Panel (**A**)) and SARS-CoV-2 spike protein (Panel (**B**)). The color and size of the circles correspond to the pairwise Pearson correlation coefficient. Data were generated by whole-blood testing of n = 30 healthy donors obtained through a blood collection protocol. The serological data are presented in Supplementary Figure S3.

Next, we sought to determine the correlation between serological and cellular responses of whole blood stimulated in vitro with SARS-CoV-2 antigens (Figure 5). No significant correlations were observed between antigen-specific cellular and serological responses when applying a multiple testing-adjusted significance threshold (α = 0.05/10 = 0.005), suggesting that assessing immunity based on cellular responses is beneficial and complementary to performing serological tests.

### 3.7. Longitudinal Changes in the Landscape of Epitope-Specific Cellular Responses

**Figure 6:**
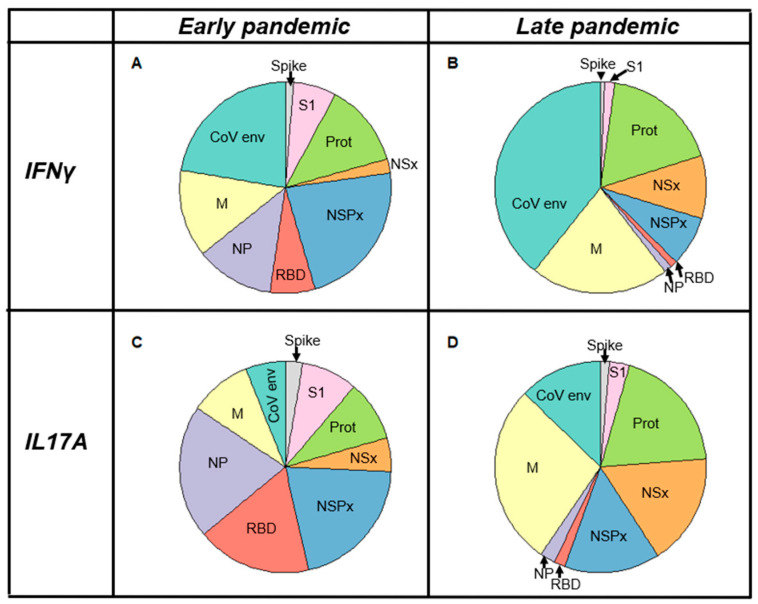
Comparison of longitudinal changes in the CoV-2-specific landscape of cytokine responses. Pie charts display the map of antigen-specific IFNγ (Panel (**A**,**B**)) and IL17A (Panel (**C**,**D**)) responses. PBMCs collected early (Panel (**A**,**C**)) vs. later on (Panel (**B**,**D**)) in the pandemic were stimulated with the indicated antigens: CoV-2 envelope (turquoise), CoV-2 membrane (yellow), CoV-2 nucleocapsid (purple), RBD (red), pool NSP peptides (blue), pool NS peptides (orange), CoV-2 protease (green), spike S1 (lilac), and CoV-2 spike protein (gray). Sample size: early-pandemic (n = 5); late-pandemic (n = 36). The accompanying raw cytokine data proportional to the data of the pie charts are provided in Supplementary Figure S4.

Immunodominant antigens are likely the main targets of cellular and serological responses. A single exposure (infection or vaccination) i expected to result in responses to immunodominant antigens while repeated viral exposure may allow the expansion of T cell clones that are specific to less immunodominant epitopes. We applied the multiplex assay to assess potential changes in the landscape of cellular responses to CoV-2-specific antigens (Figure 6). Details on the statistical analysis relating to the pie charts are provided in the Materials and Method section. Comparing the early vs. late pandemic IFNγ responses demonstrates that responses to the envelope protein (COVE) were higher later on in the pandemic. Interestingly, responses to the nucleocapsid and the RBD were more pronounced in the PBMCs from early-pandemic time points. IFNγ responses to S1 and SCOV2 were not pronounced at either time point, suggesting low immunogenicity compared to the other tested antigens.

Comparing the IL17A responses of PBMC early on vs. later on in the pandemic also revealed a shift in the dominant antigens over time. While early on in the pandemic (Figure 6C), the strongest responses were directed against the nucleocapsid and RBD (Figure 6D), responses have since shifted, with the CoV-2 membrane and env representing the larger proportions of the response, indicating a refocusing of cellular responses toward these antigens. Late-pandemic IL17A responses indicate a proportional reduction in the responses against the nucleocapsid and RBD. Responses to nonstructural proteins (NSP, NS) remained relatively stable across both time points. Like the evolution of CoV-2- specific IFNγ responses, the contribution of S1 and SCOV2 remained relatively low. The magnitude of responses to the various plate antigens between early- and late-pandemic time points was statistically highly significant in all cases (*p* < 0.01, two-proportion, two-tailed Z-test). The present data suggested that immunity to SARS-CoV-2 may evolve over time.

**Figure 7:**
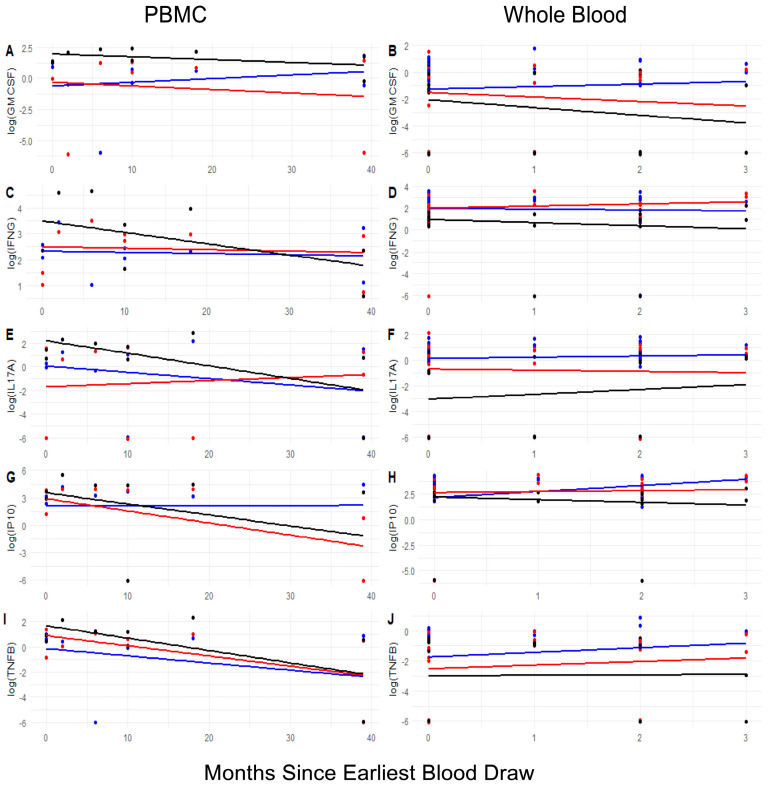
Population level-changes in cellular responses in PBMCs and whole blood over time. Scatterplots visualize changes in the magnitude of cytokine responses in PBMCs (Panels (**A**,**C**,**E**,**G**,**I**)) or whole blood (Panels (**B**,**D**,**F**,**H**,**J**)). Cytokine responses (GM-CSF: Panels (**A**,**B**); IFNγ: Panels (**C**,**D**); IL17A: Panels (**E**,**F**); IP10: Panels (**G**,**H**); TNFβ: Panels (**I**,**J**)) expressed as pg/mL were log10-transformed. Data points represent individual donors at a given time point (n = 34). Colors correspond to data points and trend lines for the stimulants: SARS-CoV-2 nucleocapsid (blue), spike protein (red), and NSPX megapool (black). Curve fitting metrics given in Supplementary Table S3.

Lastly, we sought to address the question about the persistence of CoV-2-specific cellular responses. Longitudinal changes in antigen-specific cytokine responses were plotted to determine the impact of time on the test results (Figure 7). It should be noted that the data represent population-level cross-sectional trends rather than longitudinally matched measurements for each individual.

Based on the donors’ COVID-19 disease history, we sought to determine the impact of time on the magnitude and profile of the cytokine responses (Figure 7). IL17A responses of PBMCs after in vitro stimulation with either SCOV2 or NSPX decreased over time while responses to COVN slightly increased. In contrast, stimulation of whole blood resulted in either stable responses (SCOV2 and COVN) or slight increases (CoV-2 NSPX). IFNγ responses after stimulation with SCOV2 or COVN remained stable in both PBMCs and whole blood, while responses to NSPX decreased over time in PBMCs and whole blood. Taken together, the longitudinal changes in the responses suggest that responses measured in whole blood may be more persistent.

## 4. Discussion

The present study describes the development and application of a whole-blood-based assay for assessing SARS-CoV-2-specific immunity and informing individuals on their actual COVID-19 status and history. Despite continued efforts to understand immunity to SARS-CoV-2, there are still many unanswered questions surrounding what encompasses protective immunity to SARS-CoV-2, how continuously emerging variants drive immunity, and which factors determine the susceptibility of immune individuals to new variants. The phenomenon of long COVID remains poorly understood, and reliable immune assays that capture cellular responses may be essential for uncovering its underlying pathology and aiding in diagnosis of long COVID in patients presenting with any of the wide array of symptoms but unaware of a prior mild/asymptomatic COVID-19 infection. Emerging evidence suggests that some symptoms may be linked to persistent infection or the presence of viral reservoirs [20,21,22]. While not a unique condition, long COVID may serve as a model for studying other syndromes with overlapping symptoms—such as chronic fatigue syndrome (ME/CSF)—that also lack a clearly defined etiology.

SARS-CoV-2 was the third pandemic caused by a human coronavirus, following the limited outbreaks of SARS-CoV1 and MERS. Given this pattern, future coronavirus outbreaks remain a high possibility, and thus, there is an urgent need for well-established cellular assays that are readily available. The COVID-19 pandemic highlighted how delays in deploying diagnostic assays can impact the effectiveness of public health responses and efforts to contain emerging pathogens. The persistence of long COVID across ages and genders even in the setting of more recent variants and broad population levels of immunity highlight the need for the better characterization and phenotyping of immune response to COVID-19 [3,7,8,9].

The current test is based on whole blood and, therefore, presents several advantages over testing responses based on PBMCs. The obvious advantages of testing whole blood vs. PBMCs include logistical issues with the latter, such as the requirement to isolate PBMCs prior to initiating the test. This step requires additional laboratory equipment and expertise not necessarily present in clinical testing laboratories, and it increases the cost of—and variability in—assay performance. Testing whole blood also prevents technical bias associated with PBMC isolation, such as the exclusion of higher density blood cells (granulocytes, erythrocytes), but also potentially immunomodulatory cell types such as platelets. Also lost in PBMC preparations are serum components that have been shown to play an important role in T cell activation, optimal cell activation, and immune modulation. For example, serum factors contribute to exercise-induced immunomodulation [23] and the modulation of TREM and APOE in monocyte functional assays of late-onset Alzheimer patients [24], and autologous albumin impacts humoral responses [25]. The growing body of literature indicates that testing in autologous sera may be necessary to capture immune responses that truly reflect those occurring in the respective donors [26,27]. Maintaining an autologous system has been shown to increase the accuracy of predicting in vivo responses [28] since serum factors present in the blood modulate the activation of innate and adaptive immune cells [25,27]. The novel blood test presented here retains all autologous factors and cell types and, therefore, is distinct from other existing assays (reviewed in [13]). To date, there have only been two studies comparing test results for PBMC vs. whole blood assays: (1) a tuberculosis test study demonstrated that whole blood was more sensitive and mimicked human blood conditions [29]; (2) an asthma related transcriptomics study concluded that testing whole blood resulted in higher resolution [30].

An additional advantage of testing whole blood over PBMCs became evident in our study when evaluating longitudinal changes to assess the durability of cellular responses against CoV-2 (Figure 7). In PBMC testing, dynamic changes in cytokine responses over time following infection revealed either a decline or a persistent signal, depending on the stimulating antigen. This observation aligns with findings from two other studies on the persistence of immune memory [12,31]. One of these studies specifically reported differences in immune memory persistence at the level of B cells and T cell subsets [31]. In contrast, testing whole blood demonstrated that cytokine responses to stimulation remained stable over time or even showed a slight increase (Figure 7). This finding supports conclusions from a recent review highlighting the benefits of whole-blood testing for diagnostic purposes, biomarker identification, and evaluating therapeutic outcomes [32]. To conclude, using whole blood minimizes the risk of testing errors, offering valuable insights into an individual’s potential reactions.

The newly developed assay described here quantifies cytokines produced by leukocytes after stimulation with COVN, SCOV2, or NSPX megapool in a multiplexed fashion. Assessing immunity based on more than one factor provides higher resolution and better assay performance. A recent study demonstrated the power of profiling cytokine responses and generating clusters of cytokines indicative of disease status and, therefore, of diagnostic value [33]. Similarly, longitudinal changes in the cytokine profile of COVID-19 patients mirrored progression to severe disease further emphasizing the value of assessing more than a single cytokine [34]. In the present study, we initially quantitated 54 different cytokine/chemokines produced by whole blood vs. PBMCs in response to SARS-CoV-2 antigen stimulation (Figure 1), showing that 14 of these factors were notably increased in an antigen-specific manner. The agreement of whole blood vs. PBMCs was good (r^2^ = 0.554; Figure 2A) even though there were quantitative differences in the cytokine concentrations between the two cell preparations. Down-selecting the most useful/predictive factors that would be used as readouts for the assay prototype, we chose IFNγ, IL17A, GM-CSF, TNFβ, and IP10 for further assessment and their ability to accurately classify a specimen regarding the COVID-19 history of the donor (Figure 3). Based on this data, we concluded that the assay prototype for a diagnostic test should, at a minimum, include IFNγ, IL17A, and GM-CSF. While IFNγ and IL17A are associated with responses by distinct T cell subpopulations (i.e., Th1 Vs. Th17), including GM-CSF in the panel provides the ability to assess responses beyond T cell subsets such as dendritic cells, monocytes, mast cells and NK cells after activation [35]. Data presented here (Figure 3) demonstrates that these three factors together provide the best prediction of CoV-2 immunity in the test sample. To date, the available cellular COVID-19 tests are restricted to IFNγ [2,11,12], making them less robust and less predictable.

The initial screening of SARS-CoV-2 antigens, including non-structural proteins, CoV-2 membrane, CoV-2 envelope, full-length CoV-2 spike, Spike S1 domain, RBD, COVN, NSPX, NS megapools, and SCOV2, revealed a shifting landscape of IFNγ and IL-17A responses over time (Figure 6). Our data on this limited dataset suggest that as the pandemic progressed, repeated exposures—although not always resulting in infection—boosted the precursor frequency of T cells targeting less immunogenic epitopes. This ultimately led to measurable or increased cytokine responses against specific antigens. Consequently, the contributions of anti-NSPX, RBD, COVN, and SCOV2 to the total anti-CoV-2 IFNγ response decreased, while the contributions of COVM, COVE, and NSx increased. Similarly, for IL-17A responses, the contributions of anti-RBD, COVN, and SCOV2 diminished, whereas those of anti-COVM, COVE, NSPX, NSx, and anti-CoV-2 protease increased. The differences in the fine specificity of IFNγ and IL-17A responses at various time points underscore the importance of assessing both T cell-derived cytokines to determine an individual’s COVID-19 status. A recent study on the dynamics of SARS-CoV-2-specific B and T cell subsets revealed two key findings: (1) CD4^+^ and CD8^+^ subsets exhibit distinct fine specificities, with CD4^+^ cells more frequently targeting the spike protein and CD8^+^ cells focusing on COVN; (2) T cell subsets differ in their rates of decline following an infection [31]. Consequently, the observed changes in the fine specificity of the response may also result from dynamic shifts in the persistence of different functional lymphocyte populations.

To evaluate the usefulness of the developed assay in identifying the COVID-19 status of donors, we tested 30 whole-blood samples and compared the results to the donors’ self-reported status (i.e., previously vaccinated and/or infected). Eight out of ten individuals tested in our study reporting never having had COVID-19 indeed seroconverted to COVN (Supplementary Figures S2 and S3) and had cellular responses to COVN (Figure 4), indicating prior infection. This finding highlights the value of the newly developed assay as a tool for identifying previous infections. A positive COVID-19 test result using this assay will play a vital role in diagnosing symptoms potentially associated with long COVID, a condition characterized by over 200 diverse and often challenging-to-diagnose symptoms [36].

Lastly, we reported only a weak correlation between CoV-2-specific serology and cellular responses in whole blood (Figure 5). The persistence of CoV-2-specific antibodies as an indicator of immunity or previous infection cannot be guaranteed, as their half-life depends on factors such as the individual’s age and overall health [37]. The findings of this study highlight that evaluating immunity through whole-blood cellular responses offers insights that serological tests may not reveal, underscoring the significance of the developed test and its potential as a valuable diagnostic tool.

Future studies analyzing a larger sample size in diverse populations are needed. The current study relied on samples from commercial sources and blood collection protocols that do not provide information on ethnicity, age, and any underlying health conditions. Another aspect to address is the performance of the assay for donors with comorbidities and how this will impact the cytokine profiles. Nevertheless, the presented data lay the foundation to plan studies aimed at assessing the performance of the assay in diverse populations and/or different age groups. Moreover, the assay would be a valuable tool to conduct epidemiological surveillance studies to determine prevalence in various populations. Expanding the testing to more diverse populations will help assess the reliability of the current analysis algorithm, which was developed using at least three cytokines. This will reveal whether the algorithm remains effective across different groups or requires modifications to maintain accuracy. Lastly, these studies will assist in validating the potential applications of the assay, whether for diagnosing long COVID-related symptoms or evaluating factors like susceptibility and the need for booster vaccinations.

## 5. Conclusions

We developed a simple, reliable, high-throughput assay to assess cellular responses to SARS-CoV-2, which delivers actionable results through an integrated, open-source R-based analysis pipeline. There is a growing precedent for multiplex cytokine testing in clinical contexts, including sepsis [38] and cytokine release syndrome following CAR-T therapy [39,40], where cytokine signatures have been associated with disease severity and prognosis [34,41]. Its multiplexed design and open platform enable rapid adaptation for novel pathogens, supporting clinical applications where cytokine profiling informs prognosis and treatment.

## Figures and Tables

**Table 1:** Samples analyzed throughout assay development.

Sample Cohort	Collection Period	Number of Donors
Pre.COVID	2012–2019	10
Healthy (no documented COVID-19) ^$^	2020–2022	8
COVID convalescent ^$^	2020–2024	5
Longitudinal samples ^$^	2020, 2021	34
Whole blood (healthy donors)	2023–2024	30

^$^ Positive COVID-19 history based on records of a positive COVID-19 test (PCR based).

**Table 2:** Summary of test antigens.

Antigen	Source	Concentration in Assay
SARS-CoV-2 Spike protein (Wuhan HU-1) (SCOV2) SARS-CoV-2 Receptor binding domain (RBD, COVR) HU-1	WRAIR WRAIR	5 μg/mL 1.5 μg/mL
HuCoV Spike protein 229E HuCoV Spike protein HKU-1	Sinobiologics Inc. (Houston, TX, USA) Sinobiologics Inc.	5 μg/mL 5 μg/mL
SARS-CoV-2 Nucleocapsid (COVN)	Sinobiologics Inc.	5 μg/mL
SARS-CoV-2 Envelope protein (COVE)	Sinobiologics Inc.	5 μg/mL
SARS-CoV-2 Membrane protein (COVM)	Sinobiologics Inc.	5 μg/mL
SARS-CoV-2 Protease protein (PROT)	Sinobiologics Inc.	5 μg/mL
SARS-CoV-2 ORF3A protein SARS-CoV-2 AP3A peptide pool	JPT Peptides Inc. (Berlin, Germany) JPT Peptides Inc.	1.5 μg/mL 1.5 μg/mL
**SARS-CoV-2 Nonstructural protein peptide mixes** **NSPX megapool (NSP1–NSP16)** **NSx megapool (NS6, NS7A, NS7B, NS8)**	JPT Peptides Inc. JPT Peptides Inc.	1.5 μg/mL 1.5 μg/mL
H1N1 hemagglutinin peptide pool (reference control)	JPT Peptide Inc.	1.5 μg/mL
Anti-CD3 (positive control)	Mabtech Inc. (Cincinnati, OH, USA)	1:1000
Culture medium (negative control)		NA

## Data Availability

The data are contained within the manuscript. A detailed assay protocol can be made available upon request from the corresponding author.

## Supplementary Materials

The following supporting information can be downloaded at https://www.mdpi.com/article/10.3390/jcm14196889/s1, Table S1: Accuracy of factors predicting COVID-19 status. Data express RF model accuracy of a sub-set of data (Figure 3) containing combinations of GM-CSF, IFNγ, and/or IL17A stimulated with COVN, NSPX, and/or SCOV2. Table S2: Differences in variability of serological responses to CoV-specific plate antigens stratified by previous COVID-19 disease vs. no previously recognized disease. Values expressed as log10-transformed mean luminescence signal (MLS). Table S3: Population level-changes in cellular responses in PBMC and whole blood over time. Metrics of curve fitting. Top: response to SARS-CoV-2 nucleocapsid, Middle: responses to receptor-binding domain of CoV-2 spike protein, bottom: responses to NSPX megapool. Figure S1: Variable importance plot.; The bar graph indicates the importance of the respective cytokines induced by stimulating with either COVID Nucleocapsid (CovN or COVID Spike (Scov2) in accurately predicting COVID status expressed as the Mean Decrease in Gini Impurity (MDI). Figure S2: Magnitude of antibody response to SARS-CoV-2 nucleocapsid to confirm COVID-19 status of blood donors. Data expressed as log10-transformed mean luminescence signal (MLS) specific to COVN. Red dashed line indicates cutoff value indicating seroconversion. The category of subjects (n = 30) was based on their self-assessed COVID-19 history: “negative” refers to no known previous COVID-19, “positive” refers to previous COVID-19 infection, “vaccinated” are recipients of CoV2-vaccine. Figure S3: Serological CoV-specific profiles. Results from multiplex testing were log10 transformed and stratified based on previous self-reported infection (red) vs. no self-reported infection (black) infections. Data from n = 30 donors. Antibodies were tested against indicated plate antigens (analytes): spike proteins of CoV2, CoV1, MERS-CoV, HKU-1 and OC43, SARS-CoV2 nucleocapsid, CoV2 receptor binding domain of the spike protein, CoV2 spike N-terminal domain, and hemagglutinin of H3 (Hongkong strain; used as reference analyte). Outliers were defined as points that lie outside of the interval bounded by the first quartile minus 1.5 times the interquartile range and the third quartile plus 1.5 times the interquartile range. These bounds were computed using the log10 values. Figure S4: Longitudinal changes in the CoV2-specific landscape of cytokine responses. Boxplots visualize the changes in IFNγ (Panel A) and IL17A (Panel B) responses to stimulants (indicated on *x*-axis). Sample size: early pandemic (black, n = 5), late pandemic (red, n = 36). Data are the raw data used to generate the pie charts in Figure 6.

## Declarations

EDG, JRC, ESB-L are government employees. Title 17 U.S.C. § 105 provides that “Copyright protection under this title is not available for any work of the United States Government, but the United States Government”. Title 17 U.S.C. § 101 defines US Government work as “work prepared by a military service member or employee of the US Government as part of that person’s official duties”.

## Disclaimer

Material has been reviewed by the Walter Reed Army Institute of Research. There is no objection to its presentation and/or publication. The opinions or assertions contained herein are the private views of the authors, and are not to be construed as official, or as reflecting the views of the Department of the Army or the Department of Defense. The investigators have adhered to the policies for protection of human subjects as prescribed in AR 70-25. This paper has been approved for public release with unlimited distribution.

## Abbreviations

The following abbreviations were used in this manuscript:

COVE	SARS-CoV-2 Envelope protein
COVID-19	Coronavirus Disease 2019
COVM	SARS-CoV-2 Membrane protein
COVN	SARS-CoV-2 Nucleocapsid
FDA	Food and Drug Administration
HBSS	Hank’s balanced salt solution
IRB	Institutional Review Board
LOOCV	Leave-one-out-cross-validation
MSD	Mesoscale Diagnostics
NHSR	Exempt human use protocol
NSP	*Nonstructural protein peptide pools*
PASC	Post-acute sequelae of SARS-CoV-2 infection
PBMC	Peripheral blood mononuclear cells
PCR	Polymerase chain reaction
PROT	SARS-CoV-2 Protease protein
RF	Random Forest modeling
RUO	Research use only
SARS-CoV-2	Severe acute respiratory syndrome coronavirus 2
SCOV2	SARS-CoV-2 Spike protein
