# Supplementary material for: Whole-Blood Cellular Responses: A Promising Indicator of SARS-CoV-2 Immunity Compared to Serology
Source: J Clin Med. 2025 Sep 29;14(19):6889. doi: 10.3390/jcm14196889 (PMC12524812; doi:10.3390/jcm14196889)
Supplement: Supplementary file 1 [file jcm-14-06889-s001.zip › jcm-3860301-supplementary.pdf]

Supplementary Material – Zhou et al, Whole Blood Cellular Responses: A Promising Indicator of SARS-CoV-2 Immunity Compared to Serology

1 Supplementary Tables

Supplementary Table 1: Accuracy of factors predicting COVID-19 status. Data express RF model accuracy of a sub-set of data (Figure 3) containing combinations of GM-CSF, IFNγ, and/or IL17A stimulated with COVN, NSPX, and/or SCOV2.

	COVN	COVN-NSPX	COVN-NSPX-SCOV2	COVN-SCOV2	NSPX	NSPX-SCOV2	SCOV2
GMCSF	1.00	0.91	0.93	0.97	0.85	0.91	0.94
GMCSF-IFNG	1.00	0.98	0.96	0.97	0.97	0.97	0.94
GMCSF-IFNG-IL17A	1.00	0.97	0.98	1.00	0.97	0.98	1.00
GMCSF-IL17A	0.97	0.98	0.98	1.00	0.94	0.94	0.97
IFNG	0.97	0.95	0.94	0.95	0.94	0.91	0.85
IFNG-IL17A	0.97	0.92	0.97	0.98	0.94	0.94	0.97
IL17A	0.91	0.92	0.92	0.91	0.88	0.89	0.91

Supplementary Table 2: Differences in variability of serological responses to CoV-specific plate antigens stratified by previous COVID-19 disease vs no previously recognized disease. Values expressed as log10-transformed mean luminescence signal (MLS).

Plate antigen	Confirmed previous COVID-19 infection [a]	Seropositive, but no previous recognized disease [a]
Influenza H3 (HK)	6.30 ± 0.205	6.28 ± 0.362
Spike MERS	4.77 ± 0.856	4.98 ± 0.323
Spike HKU-1	5.61 ± 0.414	5.58 ± 0.352
Spike OC43	5.95 ± 0.314	6.01 ± 0.285
Spike CoV1	5.87 ± 1.48	5.62 ± 0.455
Spike CoV2	6.36 ± 1.32	6.36 ± 0.344
RBD CoV2	6.31 ± 1.06	6.29 ± 0.557
NTD CoV2	5.91 ± 1.24	5.67 ± 0.509
COVN	5.34 ± 0.998	5.78 ± 0.737

[a] Values expressed as log10-transformed mean luminescence signal ± standard deviation

Supplementary Table 3: Population level-changes in cellular responses in PBMC and whole blood over time. Metrics of curve fitting.

Responses to CoV2 nucleocapsid

Readout		Slope	R2	p-value
GM-CSF	PBMC	0.029	0.037	0.616
GM-CSF	WB	0.185	0.006	0.67
IFN	PBMC	-0.004	0.007	0.82
IFN	WB	-0.079	0.001	0.836
IL17A	PBMC	-0.053	0.068	0.495
IL17A	WB	0.095	0.002	0.795
IP10	PBMC	0.002	0	0.977
IP10	WB	0.602	0.046	0.234
TNF	PBMC	-0.056	0.091	0.428
TNF	WB	0.306	0.017	0.476

Responses to receptor binding domain of CoV2 spike protein

Readout				
GM-CSF	PBMC	-0.028	0.021	0.708
GM-CSF	WB	-0.334	0.015	0.493
IFN	PBMC	-0.005	0.007	0.827
IFN	WB	0.193	0.015	0.5
IL17A	PBMC	0.025	0.012	0.777
IL17A	WB	-0.096	0.001	0.848
IP10	PBMC	-0.132	0.237	0.183
IP10	WB	0.098	0.001	0.846
TNF	PBMC	-0.08	0.302	0.124
TNF	WB	0.234	0.009	0.602

Responses to NSPX megapool

Readout				
GM-CSF	PBMC	-0.022	0.197	0.231
GM-CSF	WB	-0.575	0.045	0.239
IFN	PBMC	-0.043	0.249	0.171
IFN	WB	-0.282	0.025	0.379
IL17A	PBMC	-0.106	0.384	0.074
IL17A	WB	0.0379	0.015	0.49
IP10	PBMC	-0.119	0.167	0.274
IP10	WB	-0.259	0.015	0.495
TNF	PBMC	-0.098	0.369	0.082
TNF	WB	0.031	0	0.945

2. Supplementary Figures

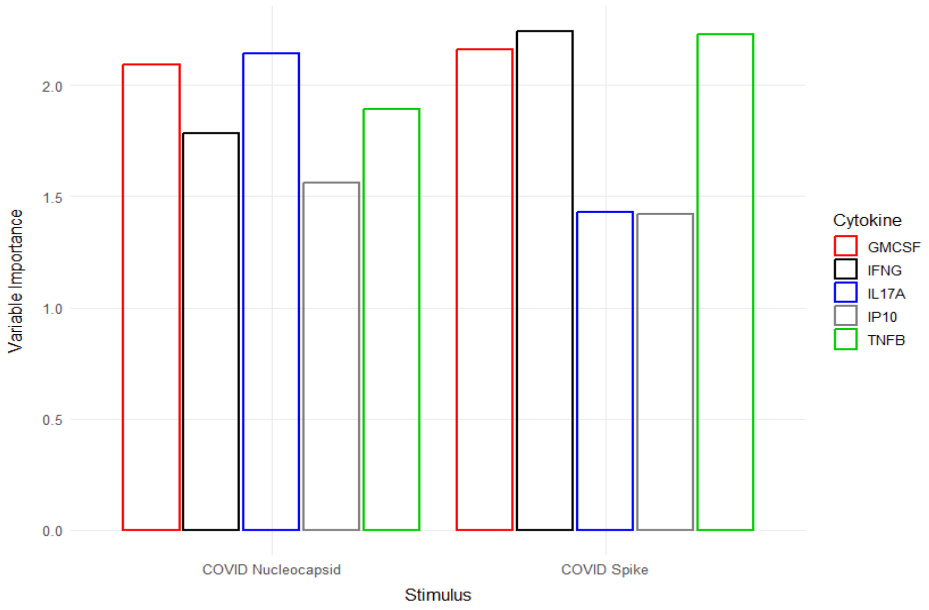

Suppl. Figure 1: Variable Importance plot. The bar graph indicates the importance of the respective cytokines induced by stimulating with either COVID Nucleocapsid (CovN or COVID Spike (Scov2) in accurately predicting COVID status expressed as the Mean Decrease in Gini Impurity (MDI).

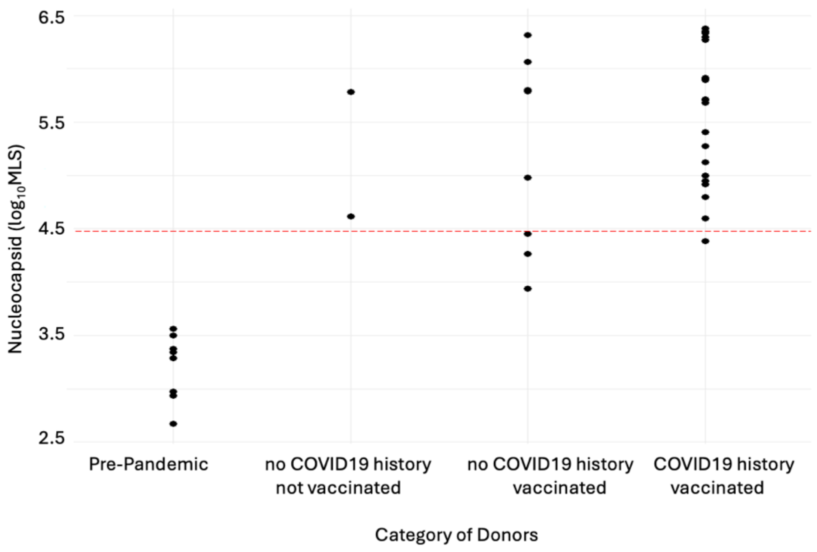

Supplementary Figure 2: Magnitude of antibody response to SARS-CoV2 nucleocapsid to confirm COVID-19 status of blood donors. Data expressed as log10-transformed mean luminescence signal (MLS) specific to COVN. Red dashed line indicates cutoff value indicating seroconversion. The category of subjects (n=30) was based on their self-assessed COVID-19 history: “negative” refers to no known previous COVID-19, “positive” refers to previous COVID-19 infection, “vaccinated” are recipients of CoV2-vaccine.

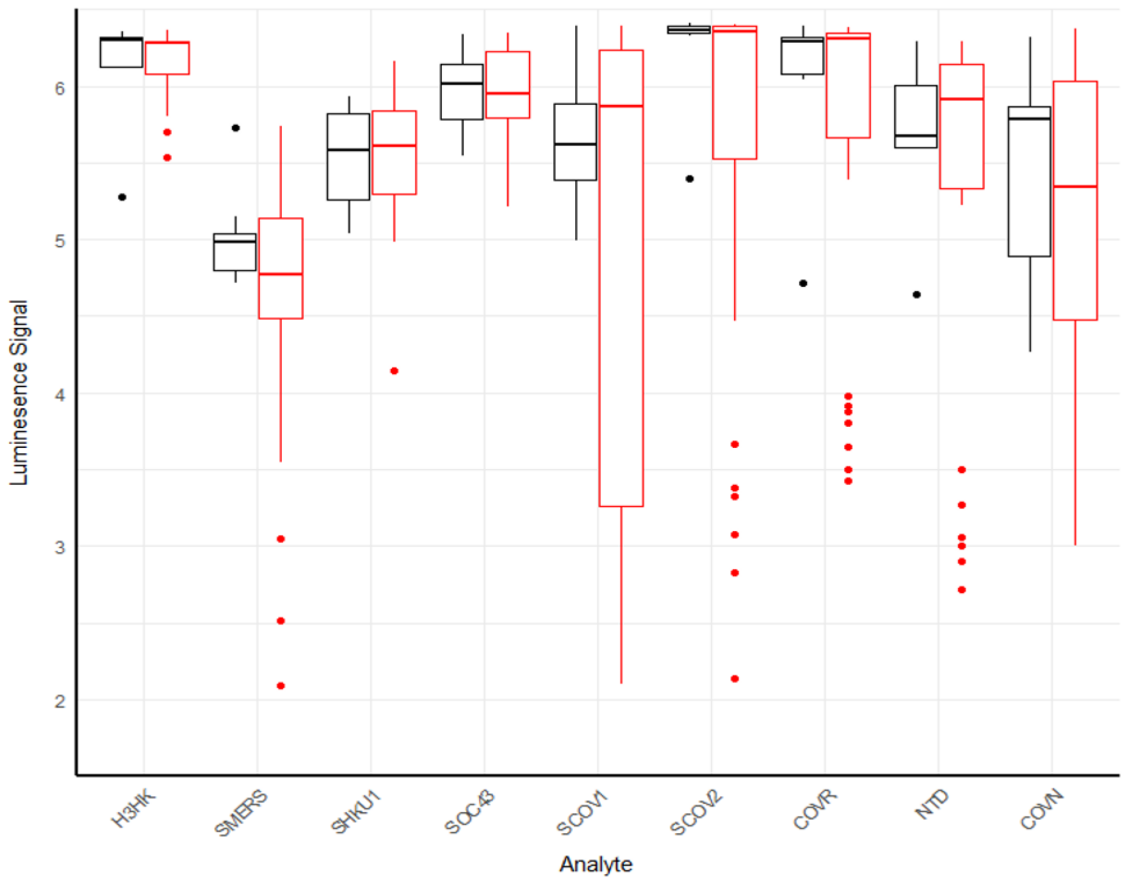

Suppl. Figure 3: Serological CoV-specific profiles. Results from multiplex testing were log10 transformed and stratified based on previous self-reported infection (red) vs. no self-reported infection (black) infections. Data from n=30 donors. Antibodies were tested against indicated plate antigens (analytes): spike proteins of CoV2, CoV1, MERS-CoV, HKU-1 and OC43, SARS-CoV2 nucleocapsid, CoV2 receptor binding domain of the spike protein, CoV2 spike N-terminal domain, and hemagglutinin of H3 (Hongkong strain; used as reference analyte). Outliers were defined as points that lie outside of the interval bounded by the first quartile minus 1.5 times the interquartile range and the third quartile plus 1.5 times the interquartile range. These bounds were computed using the log10 values.

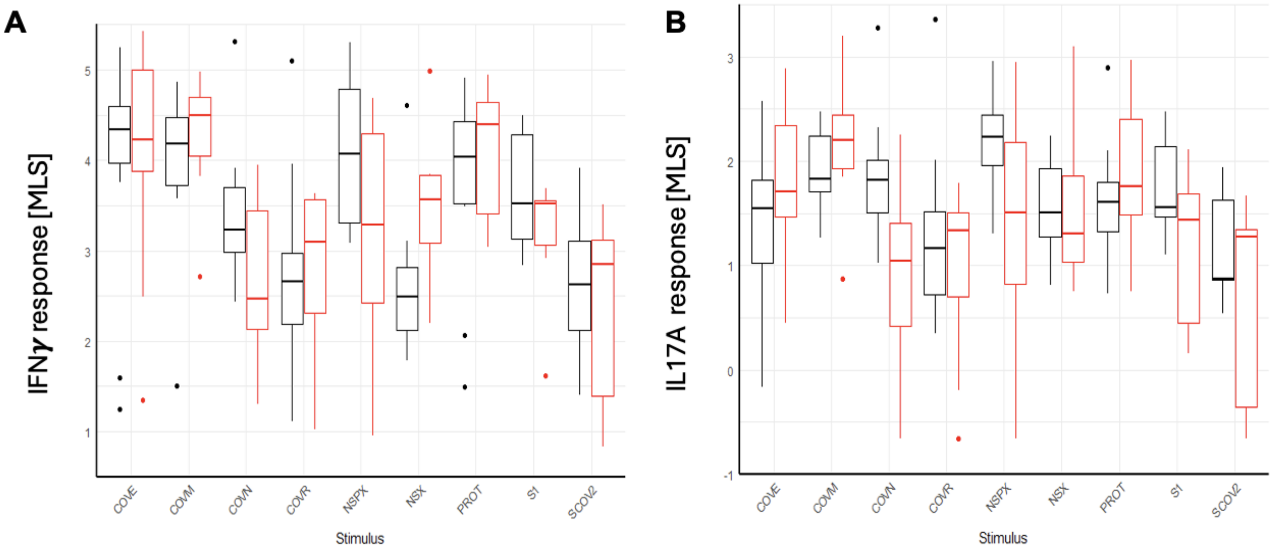

Suppl. Figure 4: Longitudinal changes in the CoV2-specific landscape of cytokine responses. Boxplots visualize the changes in IFNγ (Panel A) and IL17A (Panel B) responses to stimulants (indicated on x-axis). Sample size: early pandemic (black, n=5), late pandemic (red, n=36). Data are the raw data used to generate the pie charts in Figure 6.
